# Purification, characterization, and dehairing properties of alkaline and thermo-stable keratinase by *Penicillium citrinum* AUMC 14742

**DOI:** 10.1038/s41598-026-48471-w

**Published:** 2026-04-21

**Authors:** Osama A. M. Al-Bedak, Ayat M. A. Abdel-Latif, Nageh F. Abo-Dahab, Ahmed M. Moharram, Abdallah M. A. Hassane

**Affiliations:** 1 Assiut University Mycological Centre, Assiut, 71511 Egypt; 2 ERU Science and Innovation Center of Excellence, Egyptian Russian University, Badr City, Cairo 11829 Egypt; 3 Department of Botany and Microbiology, Faculty of Science, Al-Azhar University at Assiut, Assiut, 71524 Egypt; 4 Department of Botany and Microbiology, Faculty of Science, Assiut University, Assiut, 71511 Egypt

**Keywords:** Alkaline, Box–Behnken design, Feather, Keratinase, Purification, Biochemistry, Biological techniques, Biotechnology, Environmental sciences, Microbiology

## Abstract

The global accumulation of keratin-rich poultry waste poses significant environmental challenges due to its resistance to degradation. This study presents the first comprehensive optimization, purification, and characterization of an alkaline and thermo-stable keratinase produced by *Penicillium citrinum* AUMC 14742. The strain was confirmed morphologically and molecularly (ITS; GenBank accession PV069363) and showed strong phylogenetic placement within the *P. citrinum* clade. Keratinase production by *Penicillium citrinum* AUMC 14742 was optimized using a Box–Behnken design, which identified temperature, pH, and peptone concentration as the most influential variables. Under the optimized conditions of 26 °C, pH 8.0, and 1.5 g/L peptone, the fungus achieved a maximal keratinase activity of 312.75 U/mL. The enzyme was purified 143-fold with a specific activity of 35,423 U/mg and exhibited a molecular weight of 41.87 kDa. Biochemical characterization revealed strong alkalophilicity (optimum pH 10), thermostability (optimum 55 °C), and broad stability in the presence of metal ions, detergents, inhibitors, and solvents. Enzyme activity was markedly enhanced by Mn²⁺ (199%), Zn²⁺ (103%), Co²⁺ (101%), Tween-80 (315%), DMSO (148%), and especially 2-mercaptoethanol (≈ 1634%), while SDS, H₂O₂, Cu²⁺, and Fe²⁺ produced inhibitory effects. Kinetic analysis yielded a Km of 34 mg/mL and a Vmax of 243.9 µmol/min, indicating high catalytic efficiency toward keratin. Functionally, the crude enzyme achieved complete dehairing of goat skin within 15 h at 30 °C, demonstrating its effectiveness as an eco-friendly alternative to chemical depilation. The strong correspondence between the optimized production conditions and the enzyme’s biochemical properties highlights the robustness of the RSM model and underscores the industrial potential of this alkaline, thermo-stable keratinase.

## Introduction

Feather keratin contains high levels of cysteine, glycine, proline, arginine, and phenylalanine, which make it a valuable and rich candidate for use as a protein source^1^. The rapid expansion of global poultry processing industries has resulted in the disposal—through dumping or burning—of substantial amounts of keratin-rich waste each year, including feathers, wool, and leather^2,3^. This practice not only contributes significantly to environmental pollution but also represents a considerable loss of potentially useful biomass. Consequently, keratinous waste is now recognized as one of the most important untapped protein resources, and its effective recycling has become an urgent environmental and industrial priority^4^.

Keratin, the primary structural component of chicken feathers, is highly resistant to degradation due to its organized structure and extensive disulfide bonding, which limits susceptibility to common proteases such as trypsin, pepsin, and papain. These disulfide bonds reinforce keratin’s dense, cross-linked matrix, making enzymatic breakdown particularly challenging^5,6^. Keratinases have therefore attracted increasing attention as effective biocatalysts capable of hydrolyzing rigid keratin substrates and interacting with hydrophobic amino acid residues^7^. Their activity supports sustainable waste management by enabling the eco-friendly conversion of recalcitrant keratin waste into valuable products such as amino acids and bioactive peptides^8^. Additionally, it has the potential to enhance sustainable waste management practices while simultaneously encouraging the environmentally friendly biotransformation of resilient keratin waste into beneficial substances such as amino acids or bioactive peptides. Numerous applications of keratinase have been shown to be effective, including textile processing, leather depilation, treatment of dermatological conditions, animal feed supplements, biofertilizers, and the conversion of waste keratin into valuable materials^9,10^.

Keratinases have important industrial relevance, particularly in leather processing, where they can replace sodium sulfides in dehairing operations and enhance leather quality during tanning and beam-house procedures^11^. They also facilitate the bioconversion of keratin-rich poultry and industrial wastes into nutrient-rich proteins suitable for use in animal feed and supplements^12^. Beyond waste valorization, keratinases support the synthesis of nitrogen-based fertilizers, adhesives, films, pharmaceuticals, and biodegradable polymers. Additionally, they have been incorporated into detergent formulations to disinfect drains and launder materials contaminated with keratinous residues^13^. Owing to their substrate specificity, keratinases can efficiently clean fibrous materials without compromising their structural integrity^14^.

*Penicillium citrinum* is a widely distributed filamentous fungus and is considered one of the most frequently encountered eukaryotic organisms worldwide^15^. It has been isolated from a broad range of substrates, including soil, tropical cereals, spices, and various indoor environments^16^. This species is a consistent producer of citrinin, a nephrotoxic mycotoxin originally identified from *P. citrinum*. In addition to citrinin, the fungus is known to synthesize several other secondary metabolites, including tanzowaic acid A, quinolactacins, quinocitrinines, asteric acid, and compactin, highlighting its diverse metabolic potential^17–20^.

Recent studies indicate that keratin biodegradation primarily proceeds through two mechanisms: proteolysis and the reduction of disulfide bonds^1,6,7,21–23^. The process begins with the disruption of disulfide linkages, which exposes keratinase-accessible sites within the keratin structure. Subsequent hydrolysis of the denatured protein is then catalyzed by keratinase. Effective proteolysis requires prior cleavage of disulfide bonds to reveal susceptible regions of the substrate^8^. In this context, the present study focused on optimizing the production of a potent extracellular keratinase by *Penicillium citrinum* AUMC 14742 under submerged fermentation, followed by purification, characterization, and evaluation of its applicability in leather dehairing.

## Results

### Morphological and molecular identification of the potent strain

**Fig. 1:**
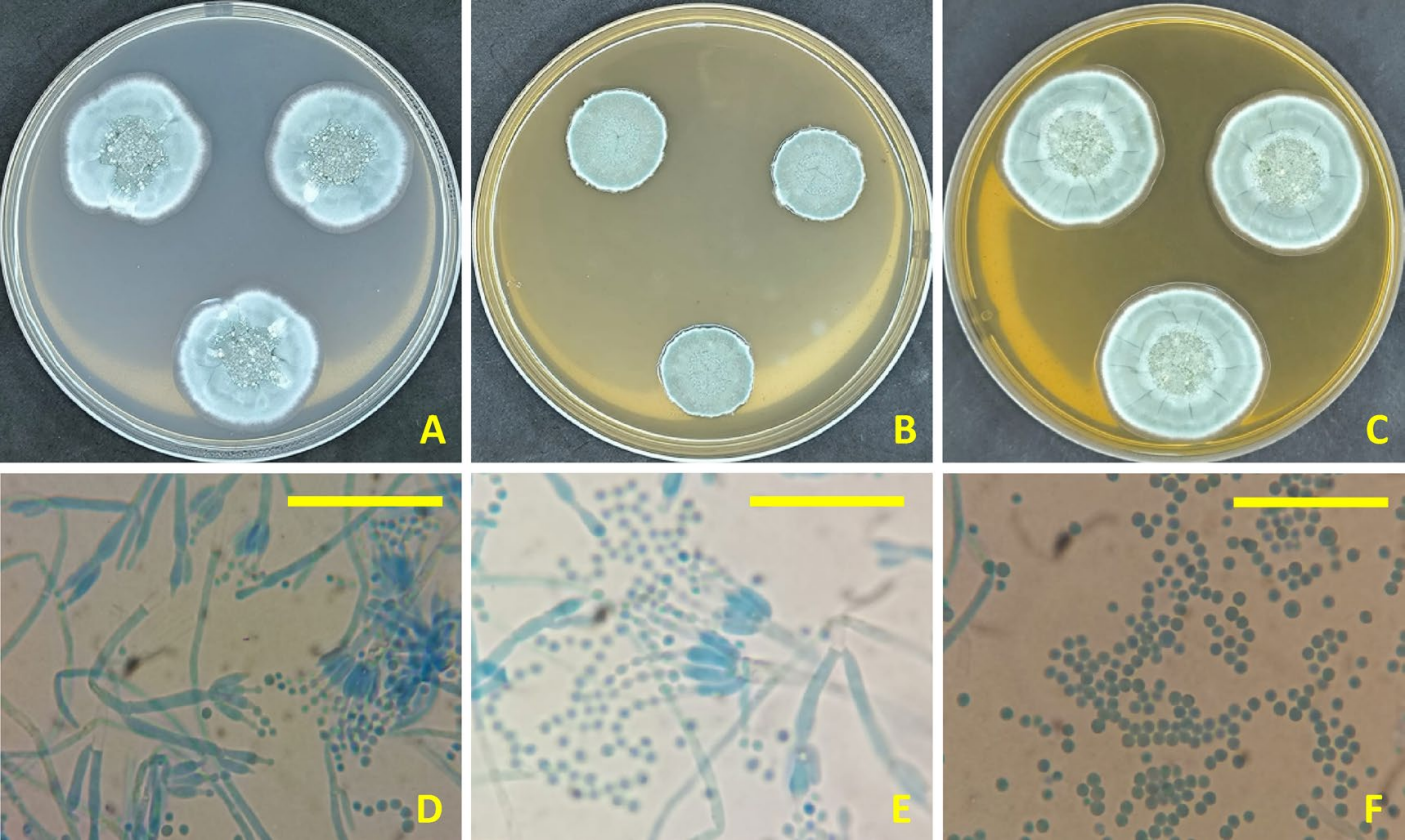
*Penicillium citrinum* AUMC 14742. (**A**–**C**) Seven-day-old colonies on Cz, MEA, and CYA at 25 °C. (**D**–**E**) Conidiophores and penicilli. (**F**) Smooth to finely roughened conidia (Scale bars = 20 μm).

The strain utilized in this study exhibited identical morphological traits to the type species *P. citrinum*. Colonies exhibited fast growth, abundant sporulation, and a grey-green coloration. Conidiophores smooth-walled, biverticillate. Metulae somewhat equal in length, 12–16 μm. Phialides ampulliform, 8–10 μm. Conidia globose to subglobose, smooth-walled, 2–2.5 μm (Fig. 1).

**Fig. 2:**
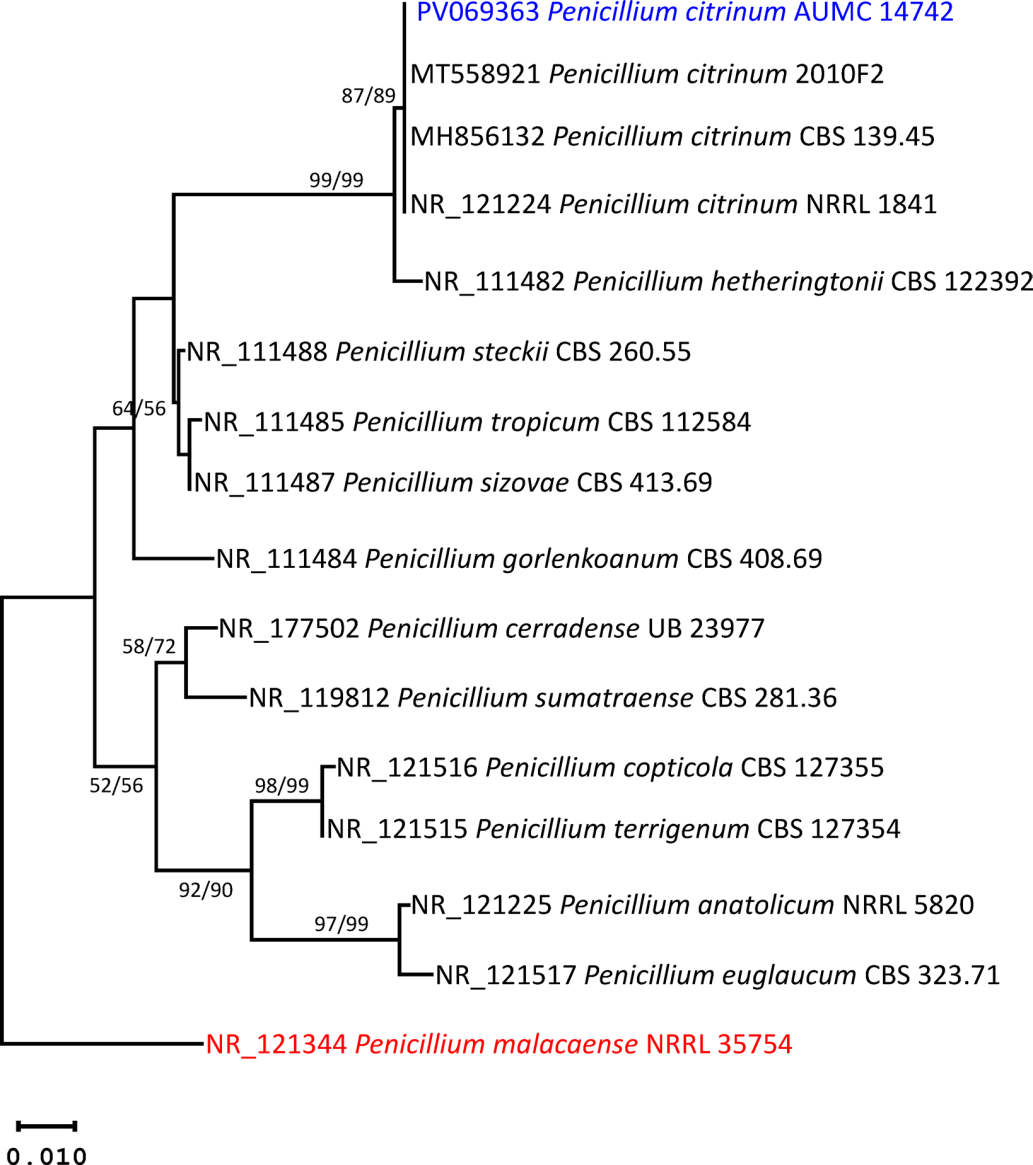
Maximum likelihood phylogenetic tree produced by ML/MP analysis using *P. citrinum* AUMC 14742 ITS sequence data (in blue) in comparison to the most comparable sequences of *Penicillium* species in GenBank. Near the corresponding nodes, bootstrap support values (1000 replications) for ML/MP ≥ 50% are displayed. Tree is rooted to *Penicillium malacaense* NRRL 35754 (in red).

Based on the ITS sequence of *P. citrinum* AUMC 14742, the closest matching strain in the GenBank database was *P. citrinum* 2010F2 (GenBank accession MT558921), showing 99.83% identity (521/522 nucleotides) with no gaps. The phylogenetic analysis demonstrated strong support for the placement of the isolate within the *P. citrinum* clade, with bootstrap values of 87% (ML) and 89% (MP). The strain clustered alongside *P. citrinum* NRRL 1841, *P. citrinum* 2010F2, and *P. citrinum* CBS 139.45 (Fig. 2), confirming its taxonomic identity as *P. citrinum*. The ITS sequence generated in this study has been deposited in GenBank under accession number PV069363 (Fig. 2).

### Optimization of keratinase production using BBD

The PBD experiment’s three most influential variables on keratinase activity— temperature, pH, and peptone—were chosen. Their optimal concentration and interaction were determined using the BBD. At run 14, the keratinase activity reached its peak (312.747 U/mL), while at run 1, it was at its lowest at 103.227 U/mL (Table 1).

**Table 1:** The BBD for keratinase production by *P. citrinum* AUMC 14742.

Run	Temperature (ºC)	pH	Peptone (g/L)	Keratinase activity
1	24	7	2	103.227
2	26	9	2	165.572
3	25	8	2	157.396
4	24	9	2	137.977
5	24	8	2.5	121.624
6	26	7	2	115.492
7	25	8	2	204.41
8	25	9	1.5	205.432
9	25	7	1.5	177.837
10	26	8	2.5	287.196
11	25	8	2	168.638
12	24	8	1.5	111.403
13	25	9	2.5	167.616
14	26	8	1.5	312.75
15	25	7	2.5	153.307

The regression equation of the BBD design for keratinase activity coded units generated using the design expert software is presented as follows (Eq. 1):

1 $$Y=172.49+50.847*A+15.842*B-9.709*C-38.681*B^2+38.995*C^2$$

Where Y represents the keratinase activity as a function of the coded levels of (A) temperature, (B) pH level, and (C) peptone concentration (g/L), respectively.

The model’s statistical significance was evaluated by ANOVA. The F-value (4.07) and p-value (0.0328) demonstrated that the chosen model was statistically significant (*p* < 0.05) for keratinase activity. The statistical model indicates a lack of interactions among the variables. This indicates that the variables are linearly independent and exert no substantial influence on one another. The F-values of individual variables indicated that temperature exerted the most significant influence, whilst peptone showed the least effect on keratinase activity (Table 2).

**Table 2:** ANOVA test results for keratinase activity.

No	Code	Source	Sum of squares	Df	Mean square	F value	*P* value
		Model	35,512.43	5	7102.49	4.07	0.0328
1	A	A-Temp	20,683.32	1	20,683.32	11.87	0.0073
2	B	B-pH	2007.69	1	2007.69	1.15	0.3111
3	C	C-Peptone	754.19	1	754.19	0.4327	0.5271
4	B²	B²	5557.29	1	5557.29	3.19	0.1078
5	C²	C²	5648.02	1	5648.02	3.24	0.1054
		Residual	15,687.87	9	1743.1		
		Lack of Fit	14,482.42	7	2068.92	3.43	0.2441
		Pure Error	1205.45	2	602.73		

*p* < 0.05 were significant R^2^ = 0.6936, R^2^ (adj) = 0.5234.

**Fig. 3:**
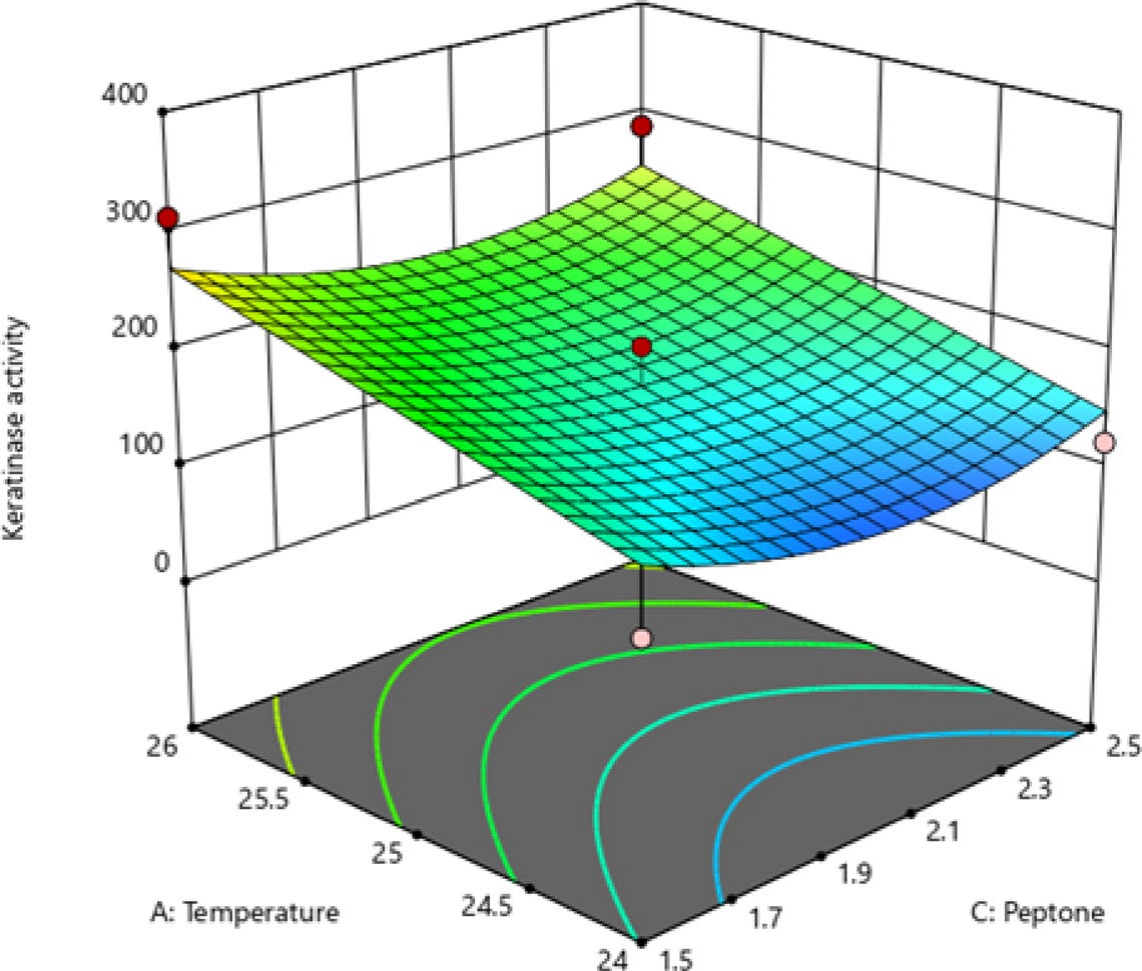
3D surface plot representing interaction effect.

Regression analysis was used to generate the 3D surface plot (Fig. 3), allowing evaluation of variable interactions and identification of their optimal levels for maximizing keratinase production. In this plot, two factors were varied while the third was maintained at its midpoint. The results indicated that both pH and temperature substantially influence keratinase activity. Under conditions of low peptone concentration (1.5 g/L) and elevated temperature (26 °C), the enzyme exhibited its highest predicted activity (273.66 U/mL).

### Validation

The objective was to identify the most effective model for enhancing keratinase activity. To ensure optimal model performance, the experimental design was refined and validated. Using the parameter set recommended by the RSM optimizer, an initial validation experiment was conducted, in which the predicted keratinase activity (273.66 U/mL) closely matched the measured value (272.82 U/mL), confirming the model’s accuracy. A second experiment was performed to further assess predictive reliability, and the observed activity (208.5 U/mL) was similarly aligned with the predicted value (223.34 U/mL), demonstrating strong model robustness (Table 3).

**Table 3:** Optimal settings of medium’ components.

Parameter settings from RSM optimizer			Keratinase	
Temperature	pH	Peptone (g/L)	Actual value	Predicted value
26	8.2	1.5	272.82	273.66
26	8	2	208.50	223.34

### Keratinase purification

*Penicillium citrinum* AUMC 14742 produced keratinase efficiently under optimized submerged fermentation conditions. The resulting crude enzyme supernatant (1,450 mL) was sequentially purified using cold absolute ethanol precipitation, MP-800 anion-exchange chromatography, and Sephadex G-50 gel filtration. This process yielded a homogeneous enzyme preparation with a final recovery of 6.5%, a 143-fold increase in purity, and a specific activity of 35,423 U/mg (Table 4).

**Table 4:** Purification profile of keratinase produced by *P. citrinum* AUMC 14742 at pH 8.0 and 26 °C after 4 days using peptone as nitrogen source in SmF.

Purification steps	Volume (mL)	Activity (U/mL)	Total activity (U)	Protein (mg/mL)	Total protein (mg)	Specific activity (U/mg)	Yield (%)	Fold
Fermentation medium	1450	312	452,400	1.26	1827	247.62	100	1
Absolute ethanol	125	760	95,000	3.65	456.25	208.22	20.99	0.84
MP 800	35	1340	46,900	1.18	41.3	1135.6	10.36	4.58
Sephadex G 50	16	1842	29,472	0.052	0.832	35,423	6.5	143

### Determination of molecular weight by SDS-PAGE

**Fig. 4:**
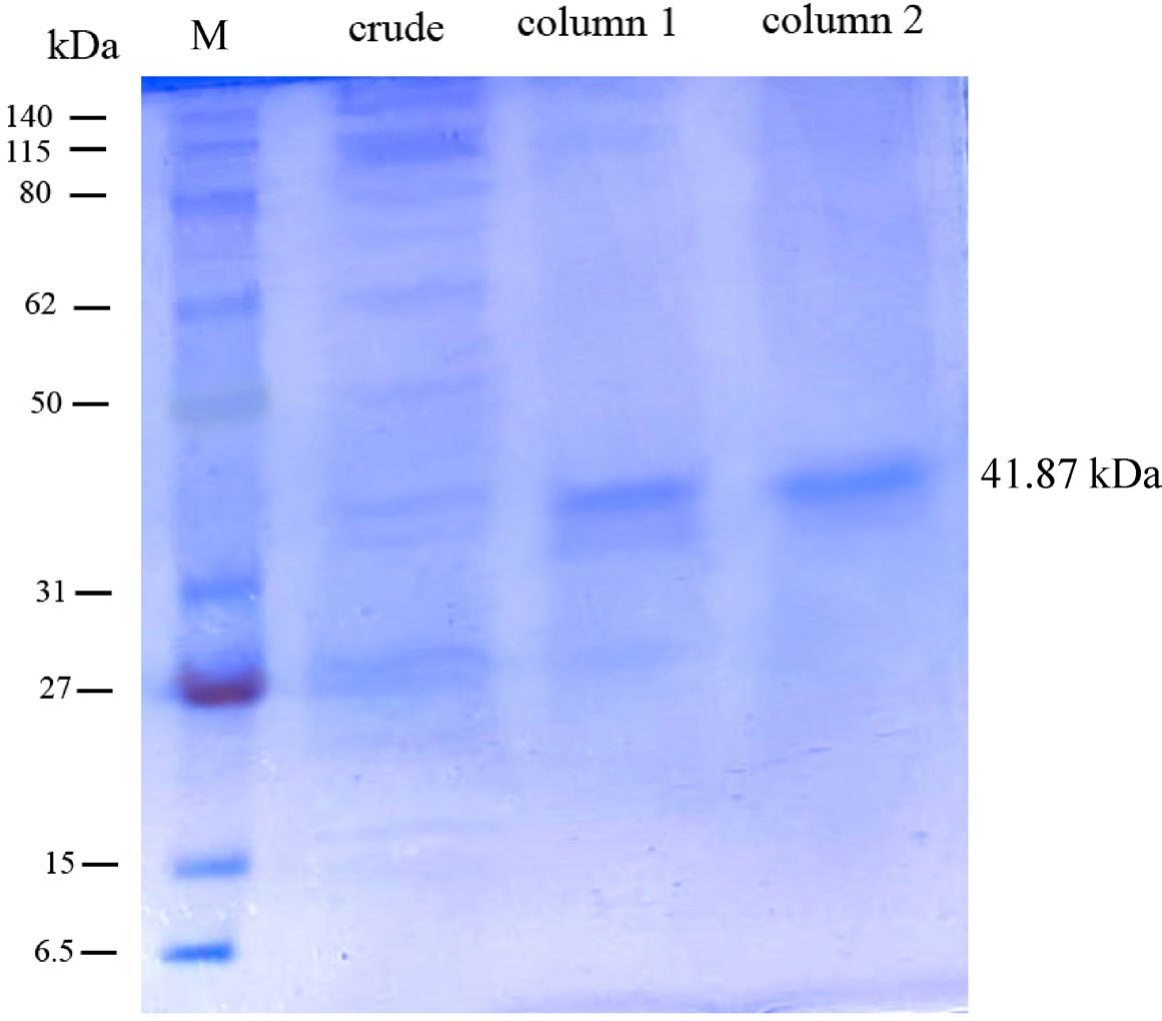
SDS-PAGE of keratinase produced by *P. citrinum* AUMC 14742 showing: Lane 1 standard marker (M); Lane 2: crude keratinase; Lane 3: MP 800 column; and Lane 4: Sephadex G 50.

SDS–PAGE analysis confirmed that the keratinase produced by *P. citrinum* AUMC 14742 was homogeneous and fully purified, with an estimated molecular weight of 41.87 kDa (Fig. 4).

### Effect of pH on the activity of purified keratinase

**Fig. 5:**
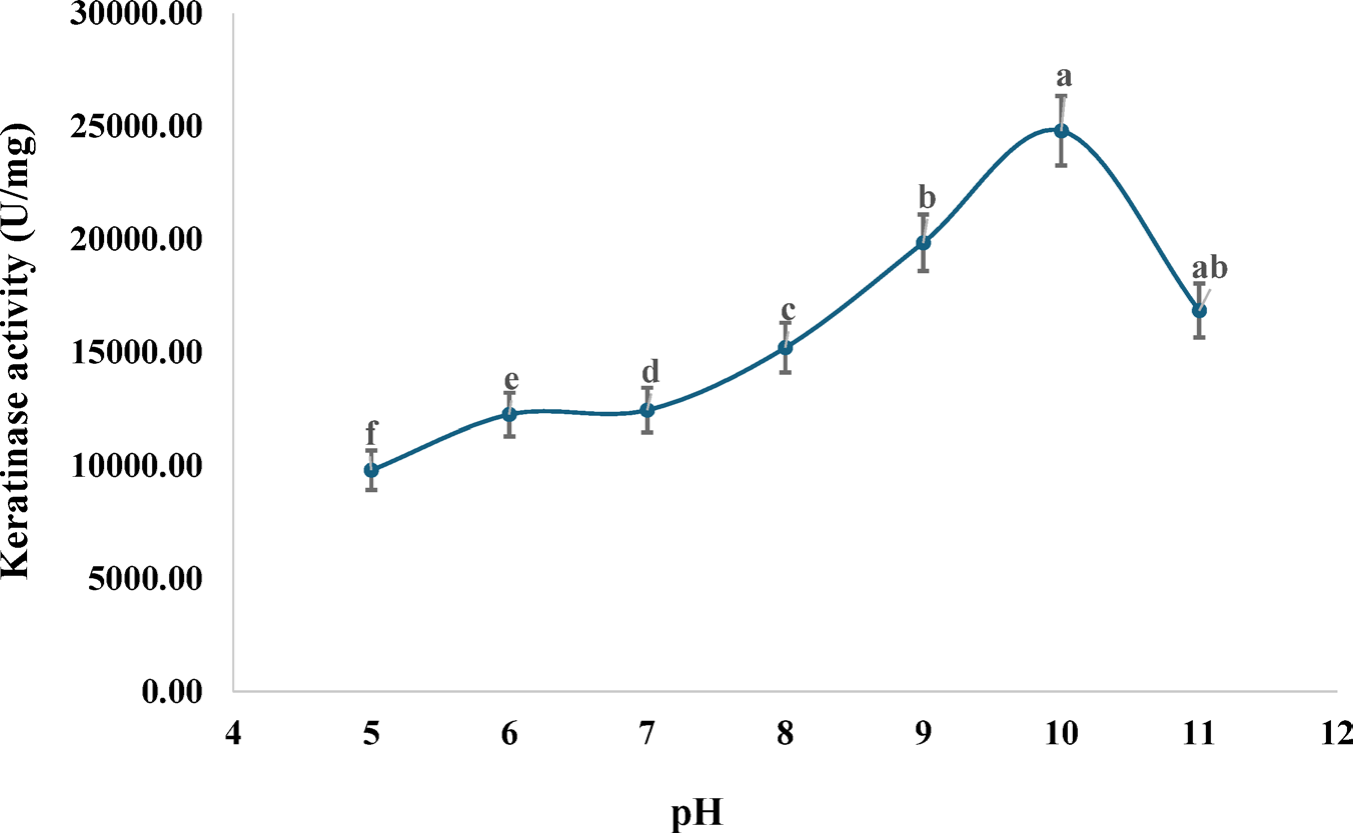
Effect of pH on the activity of pure keratinase produced by *P. citrinum* AUMC 14742 (Mean ± SD with different letters is significantly different (*p* < 0.05; *n* = 3).

The purified keratinase from *Penicillium citrinum* AUMC 14742 showed activity across a broad pH range (5–11), with a clear optimum at pH 10.0, where the enzyme specific activity reached 24,806 ± 1,540 U/mg. This indicated that the enzyme is strongly alkaline-active, a desirable feature for industrial applications such as detergents, leather processing, and feather hydrolysis, where high-pH environments are common. At pH values below or above the optimum, activity decreased, suggesting that deviations from alkaline conditions may disrupt the enzyme’s structural conformation or active-site environment (Fig. 5).

### Effect of temperature on the activity of purified keratinase

**Fig. 6:**
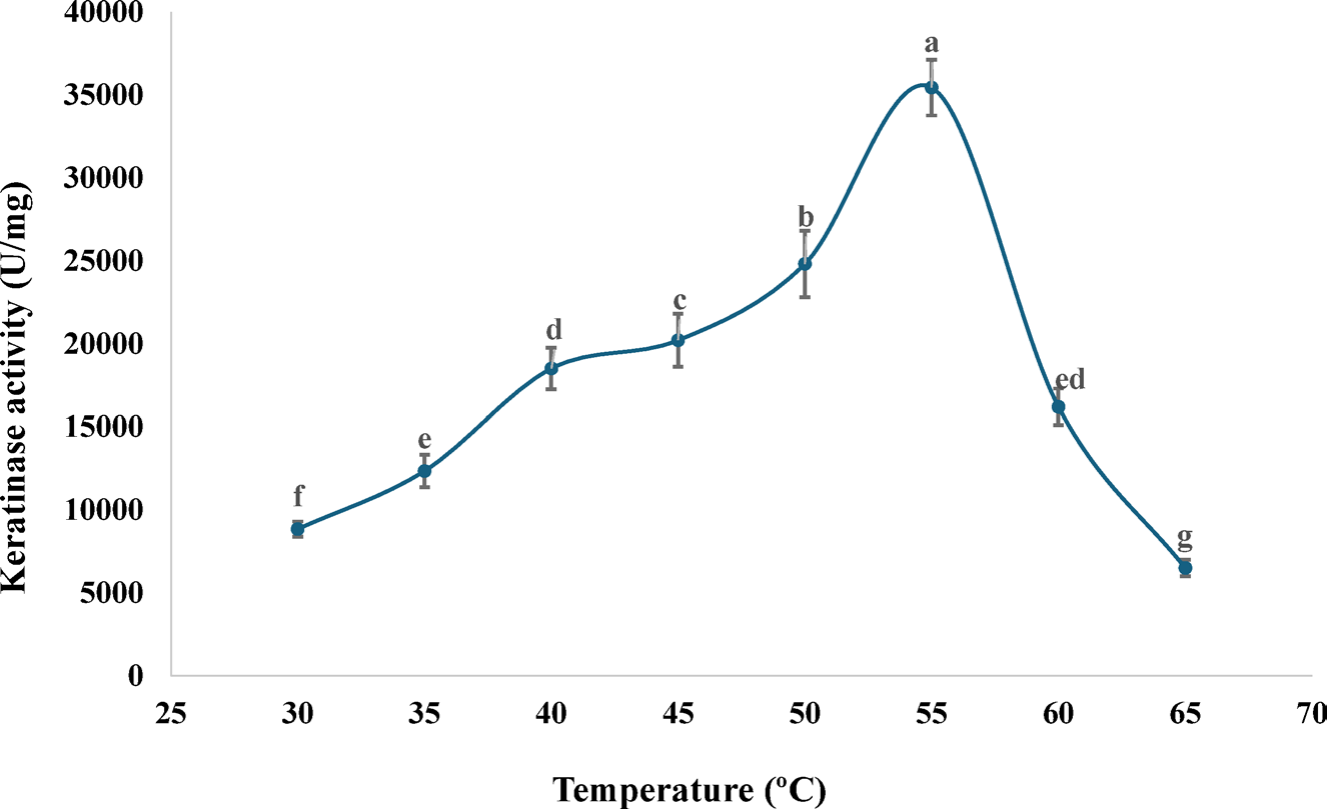
Effect of temperature on the activity of pure keratinase produced by *P. citrinum* AUMC 14742 (Mean ± SD with different letters is significantly different (*p* < 0.05; *n* = 3).

Temperature assays showed that the keratinase exhibits significant thermostability, with activity measurable from 30 °C upward. The enzyme reached its maximum activity at 55 °C, recording 35,437 ± 1684 U/mg. This thermal optimum indicated that the enzyme performs efficiently under moderately high temperatures, which is advantageous for industrial bioprocesses that rely on accelerated reaction rates or elevated operating temperatures. Beyond 55 °C, activity typically declined due to thermal denaturation or loss of structural integrity (Fig. 6).

### Effect of some chemical’s addition on the activity of pure keratinase

The influence of metal ions on keratinase activity varied considerably. Among the ions tested, Zn²⁺, Mn²⁺, and Co²⁺ markedly enhanced enzymatic activity, increasing it to 103.34 ± 3.59%, 199.16 ± 4.14%, and 101.25 ± 2.13%, respectively, relative to the control. In contrast, Na⁺, K⁺, Ca²⁺, Mg²⁺, Cu²⁺, Ni²⁺, and Fe²⁺ exerted inhibitory effects, reducing activity to 87.86 ± 3.06%, 94.14 ± 4.09%, 94.98 ± 1.56%, 90.79 ± 2.58%, 71.13 ± 3.59%, 98.33 ± 4.62%, and 79.91 ± 5.16%, respectively, compared with the control (Table 5).

**Table 5:** Effect of metal ions on the activity of the pure keratinase produced by *P. citrinum* AUMC 14742 (Mean value ± SD with different letters is significantly different (*p* < 0.05; *n* = 3).

Chemical added	Specific activity (U/mg)	Residual activity (%)
Control	35,437 ± 1684^bc^	100 ± 4.75
NaCl	31,135 ± 1084^e^	87.86 ± 3.06^e^
KCl	33,360 ± 1449^cde^	94.14 ± 4.09^cde^
CaCl_2_	33,658 ± 1552^cde^	94.98 ± 1.56^cde^
MgSO_4_	32,173 ± 914^de^	90.79 ± 2.58^de^
FeSO_4_	28,318 ± 1828^f^	79.91 ± 5.16^f^
CuSO_4_	25,206 ± 1272^g^	71.13 ± 3.59^g^
ZnSO_4_	36,621 ± 1272^b^	103.34 ± 3.59^b^
MnSO_4_	70,577 ± 1467^a^	199.16 ± 4.14^a^
CoCl_2_	35,880 ± 1754^bc^	101.25 ± 2.13^bc^
NiCl_2_	34,845 ± 1637^bcd^	98.33 ± 4.62^bcd^

### Effects of inhibitors, detergents, and reducing agents on keratinase activity

The addition of various chemicals significantly enhanced keratinase activity. EDTA, PMSF, 10% hexane, 10% methanol, 5% and 10% ethanol, 5% and 10% 2-mercaptoethanol, 5% and 10% Tween-80, and 5% and 10% DMSO increased enzyme activity to 111.71 ± 3.69%, 108.37 ± 3.59%, 125.10 ± 4.14%, 105.02 ± 3.13%, 108.78 ± 4.14%, 118.41 ± 2.58%, 1633.46 ± 4.27%, 1634.29 ± 3.69%, 310.46 ± 2.85%, 315.48 ± 4.14%, 109.2 ± 3.07%, and 148.12 ± 1.02%, respectively, compared with the control (Table 6).

**Table 6:** Effect of metal ions, inhibitors, organic solvents and reducing agents on the activity of the pure keratinase produced by *P. citrinum* AUMC 14742 (Mean values ± SD with different letters are significantly different (*p* < 0.05; *n* = 3).

Chemical added	Specific activity (U/mg)	Residual activity (%)
Control	35,437 ± 1684^fg^	100 ± 4.75
EDTA (5 mM)	39,587 ± 1307.6^e^	111.71 ± 3.69^e^
SDS (5 mM)	30,543 ± 1272^jk^	86.19 ± 3.59^jk^
PMSF (5mM)	38,403 ± 1272^e^	108.37 ± 3.59^e^
5% H_2_O_2_	25,206 ± 552.8^no^	71.13 ± 1.56^no^
10% H_2_O_2_	24,019 ± 361.5^o^	67.78 ± 1.02^o^
5% Hexane	31,284 ± 1109^ijk^	88.28 ± 3.13^ijk^
10% Hexane	44,332 ± 1467^d^	125.10 ± 4.14^d^
5% Toluene	31,730 ± 552.8^hijk^	89.54 ± 1.56^hijk^
10% Toluene	32,917 ± 1662^ghij^	92.89 ± 4.69^ghij^
5% Chloroform	30,245 ± 1449^kl^	85.35 ± 4.09^kl^
10% Chloroform	28,023 ± 726.5^lm^	79.08 ± 2.05^lm^
5% Benzene	26,390 ± 914^mno^	74.47 ± 2.58^mno^
10% Benzene	27,570 ± 1449^mn^	77.8 ± 4.09^mn^
5% Methanol	33,360 ± 1087.9^ghi^	94.14 ± 3.07^ghi^
10% Methanol	37,216 ± 1109^ef^	105.02 ± 3.13^ef^
5% Ethanol	38,548 ± 1467^e^	108.78 ± 4.14^e^
10% Ethanol	41,961 ± 914^d^	118.41 ± 2.58^d^
5% Acetone	34,250 ± 1087.9^gh^	96.65 ± 3.07^gh^
10% Acetone	34,250 ± 1449^gh^	96.65 ± 4.09^gh^
5% DMSO	38,697 ± 1087.9^e^	109.2 ± 3.07^e^
10% DMSO	52,489 ± 361.5^c^	148.12 ± 1.02^c^
5% Tween-80	110,019 ± 1009.9^b^	310.46 ± 2.85^b^
10% Tween-80	111,797 ± 1467^b^	315.48 ± 4.14^b^
5% 2-Mercaptoethanol	578,856 ± 1513^a^	1633.46 ± 4.27^a^
10% 2-Mercaptoethanol	579,150 ± 1307.6^a^	1634.29 ± 3.69^a^

### Determination of K_m_ and V_max_

**Fig. 7:**
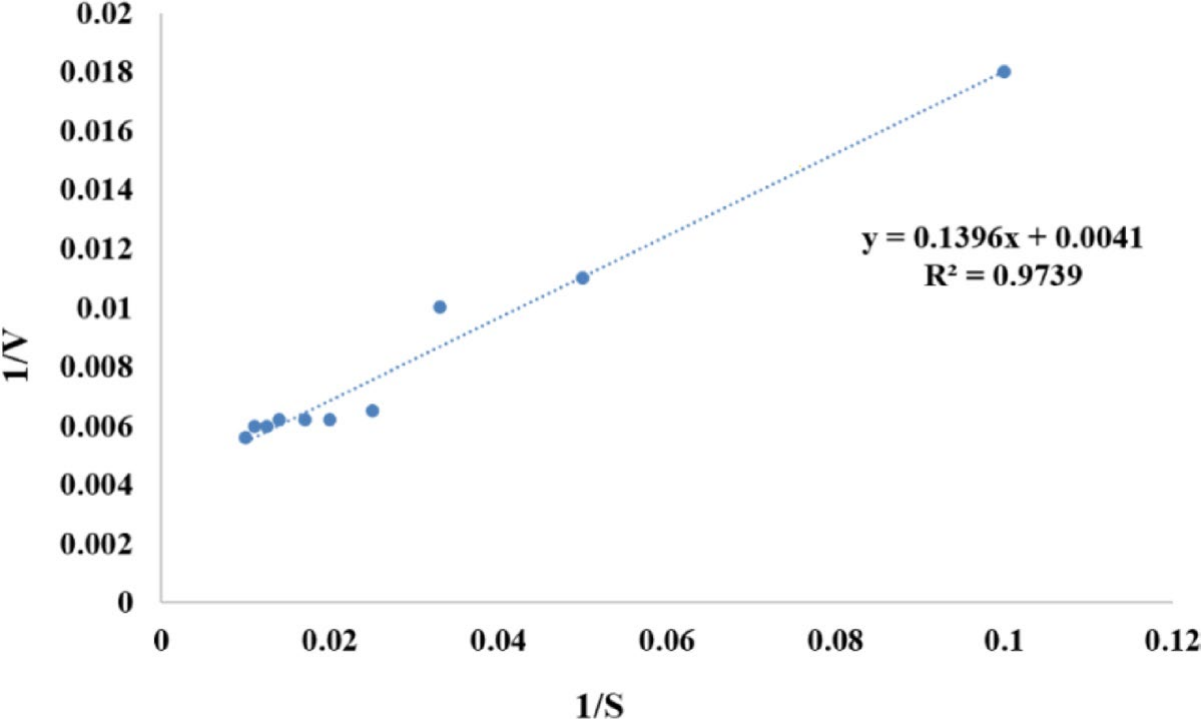
Lineweaver-Burk plot of the reciprocal of initial velocities and keratin concentration.

K_m_ and V_max_ were determined from a Lineweaver–Bürk plot to be 34 mg/mL and 243.9 µmol/min, respectively (Fig. 7).

### Dehairing activity

**Fig. 8:**
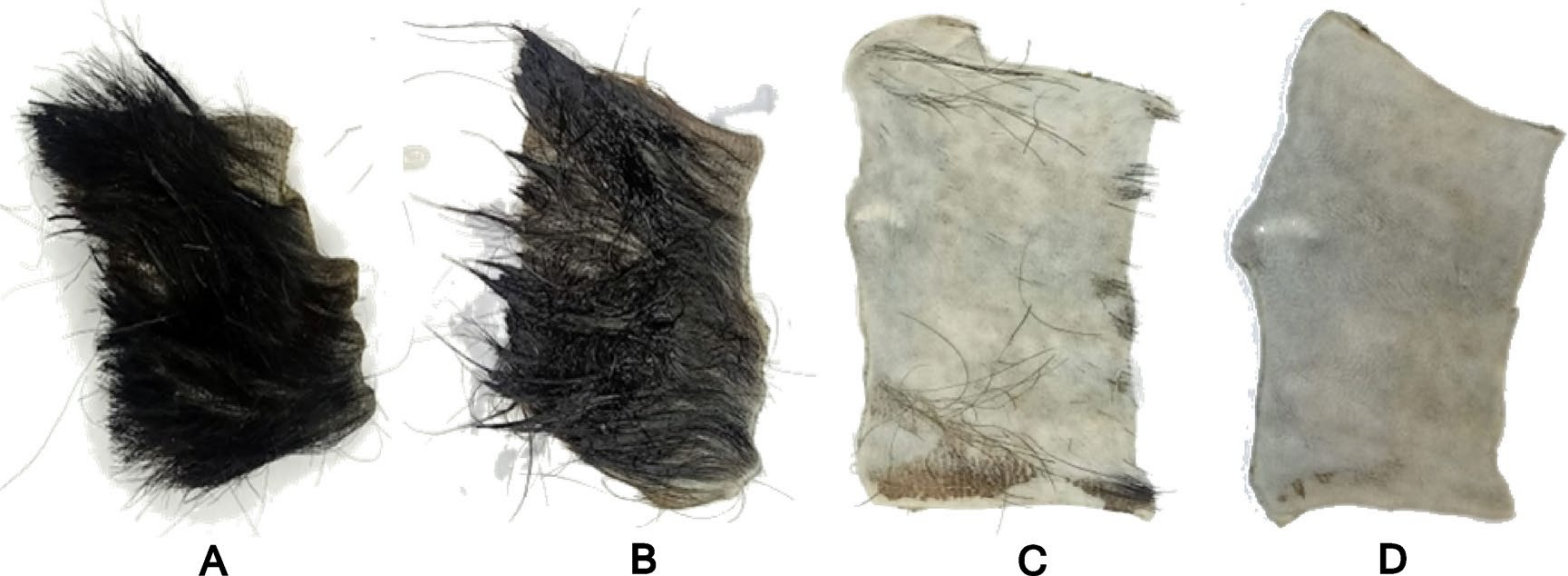
Dehairing of goat skin by crude keratinase produced by *P. citrinum* AUMC 14742. (**A**) Control skin dipped in sterile distilled water. (**B**) The skin dipped in crude keratinase preparation after 5 h of incubation at 30 °C. (**C**) The skin dipped in crude keratinase preparation after 10 h of incubation 30 °C. (**D**) The skin dipped in crude keratinase preparation after 15 h of incubation 30 °C.

Complete dehairing of goat skin was achieved after 15 h of treatment with the crude keratinase produced by *P. citrinum* AUMC 14742 at 30 °C. Following enzymatic dehairing and rinsing with distilled water, the skin appeared smooth, pliable, and free of residual hair (Fig. 8).

## Discussion

There is increasing interest in the use of keratinases for managing keratin-rich waste, as these enzymes provide a more sustainable and cost-effective alternative to conventional physicochemical treatments. Keratinase-based processing yields higher-quality by-products, minimizes the generation of hazardous chemicals, and reduces overall environmental impact^4^. Incorporating keratinase into animal feed has been shown to enhance immune response, palatability, digestibility, and beneficial microbiota in livestock^9^. Because of its high nitrogen content, keratinase-treated waste can also be converted into effective biofertilizers^24^. In addition to its use in agriculture, keratinase has shown promise in the medical and environmental fields, particularly in the treatment of prion contamination and onychomycosis, acne, and psoriasis^25^. Traditional chemical treatments, on the other hand, are associated with a number of hazards, such as the development of damaging residues, tissue damage, and overpowering unpleasant smells^26^.

The optimization of keratinase production using the Box–Behnken design (BBD) provided the experimental foundation needed to interpret the biochemical characteristics of the enzyme in a mechanistic and application-oriented context. Statistical screening first identified temperature, pH, and peptone concentration as the most influential variables governing keratinase production by *P. citrinum* AUMC 14742, with temperature exerting the strongest effect on enzyme yield. This was validated by the regression model and ANOVA analysis, which confirmed the significant contribution of temperature and the subordinate but measurable influence of pH and peptone. The BBD-generated response surface revealed that high activity was favored under moderately alkaline conditions (pH 8), elevated but non-stressful temperatures (26 °C), and low peptone concentrations (1.5 g/L), leading to a maximum determined activity of 312.75 U/mL. These optimized conditions were further corroborated through validation experiments, demonstrating the predictive accuracy and reliability of the RSM-based model. Keratinase activity is widely used as a key indicator of fungal keratinolytic potential, as demonstrated in several previous studies^27,28^. In this regard, Liang et al.^28^ demonstrated that optimal keratinase production by *Myceliophthora thermophila* at pH 7.9 using 0.98 g/L urea, while Anbu et al. reported maximal activity of *Scopulariopsis brevicaulis* within pH 6.5–7.9 and 25–47.5 °C using a Box–Behnken design^29^. Similarly, response-surface optimization increased keratinase yield in *S. brevicaulis* by 6.18-fold through adjustment of glucose, soybean meal, feather powder, and inoculum levels^30^. Additional studies have shown diverse optimal parameters among keratinolytic fungi and bacteria, including a 12.72-day optimum at 35 °C for *Curvularia lunata* JK17^31^ using 5.9 g/L peptone, and a four-day optimum at pH 7 and 40 °C for *Streptomyces* sp. DZ 06 determined through Box–Behnken analysis. These comparisons reinforce the effectiveness of statistical design tools in defining the precise conditions needed to maximize keratinase synthesis across diverse microbial systems^32^.

Ramnani and Gupta^33^ achieved a 3.5-fold increase in keratinase production from *Bacillus licheniformis* RG1 using a combined Plackett–Burman and response-surface optimization strategy. Similarly, Vigneshwaran et al.^34^ identified *B. licheniformis* as a promising keratinase producer, reporting a maximum activity of 10.76 U/mL. In the present study, *P. citrinum* AUMC 14742 exhibited optimal keratinase production when peptone was used as the nitrogen source. Previous research has identified various preferred nitrogen sources for keratinase synthesis, including ammonium chloride^35^, yeast extract and potassium nitrate^36^, and casein^37^. These nitrogen sources enhance the formation of soluble proteins and amino acids, thereby promoting enzyme synthesis and increasing the agricultural value of feather hydrolysates^13^. However, peptone has not always been beneficial; for instance, its use in *Arthrobacter* sp. NFH5 resulted in reduced keratinase yield^36^.

The enzyme production by *P. citrinum* AUMC 14742 was monitored in this study over a seven-day period to assess its keratinolytic activity in relation to fungal growth. Keratinase production and feather degradation were minimal during the first two days; however, by day four, the fungus exhibited substantial growth accompanied by maximum enzyme yield. After this point, no further increase in activity was observed, indicating that *P. citrinum* AUMC 14742 efficiently utilized feather substrate to produce keratinolytic enzymes within a relatively short timeframe. Comparable trends have been reported for other keratinolytic microorganisms, including *Myrothecium verrucaria*, which achieved peak protease production after four days in feather-based solid-state fermentation^38^., as well as *Fusarium* sp. 1 A, which required three weeks of incubation to reach maximum activity^39^. Similarly, *Bacillus cereus* displayed its highest keratinase output after 96 h of feather degradation^4^. These comparisons highlight the superior efficiency of *P. citrinum* AUMC 14742, which reaches maximal keratinase production more rapidly than several other reported strains—a feature of considerable value for industrial applications.

The biochemical characterization of the purified keratinase aligned closely with the physiological preferences identified during optimization, indicating that the production conditions favor the enzyme’s inherent catalytic nature. For example, while the enzyme is produced most efficiently at 26 °C, its catalytic optimum occurs at a higher temperature of 55 °C. This suggests that *P. citrinum* synthesizes the enzyme under growth-supportive conditions, yet the enzyme is structurally adapted to function under more thermally demanding environments. This thermostability is reflected in the high specific activity achieved after purification (35,423 U/mg) and the enzyme’s capacity to retain substantial activity well above growth temperature. Likewise, the optimal production pH of 8 corresponds to an enzyme that exhibits maximal activity at pH 10, demonstrating that the organism produces a strongly alkaline-active keratinase even when cultivated under moderately alkaline conditions. Such divergence between production and catalytic optima is a common hallmark of industrially relevant enzymes, where microbial physiology dictates optimal growth conditions while the secreted enzyme is evolutionarily adapted for performance in harsher or more specialized environments.

The effects of various chemicals and inhibitors, including metal ions, reducing agents, and organic solvents—were evaluated to understand their influence on keratinase activity and to explore potential applications based on the enzyme’s intrinsic properties. Among the tested ions, Zn²⁺, Mn²⁺, and Co²⁺ markedly enhanced enzyme activity, whereas Na⁺, K⁺, Ca²⁺, Mg²⁺, Cu²⁺, Ni²⁺, and Fe²⁺ reduced activity. The stimulatory or inhibitory impact of specific ions may reflect their roles in stabilizing enzyme structure or modulating substrate binding. For example, metal ions can function as ionic bridges that help maintain the catalytic conformation of the enzyme or strengthen enzyme–substrate interactions^40^. Consistent with this interpretation, Secades et al. reported that Ca²⁺ can stabilize the tertiary structure of microbial proteases, thereby affecting autolysis^41^. Additionally, several microbial metalloproteases use Mn²⁺ and Mg²⁺ as protective agents against thermal denaturation^42^. However, previous findings are not always in agreement with the present results; ZnCl₂ was shown to decrease keratinase activity by 64% in *A. oryzae*, and CaCl₂ showed no measurable effect^43^. Similarly, Korkmaz et al. observed that ZnCl₂ reduced keratinase activity by 18–19%, whereas CaCl₂ enhanced it^44^. These variations reflect the complex roles of divalent ions in enzyme catalysis, as many (e.g., Zn²⁺) participate in catalytic processes that can be inhibited by heavy metals, chelating agents, or transition metals—an attribute commonly associated with this class of enzymes^45^.

The keratinase produced in this study was inhibited by SDS, whereas EDTA, PMSF, and DMSO enhanced its activity. The observed lack of inhibition by EDTA may be attributed to competition between EDTA and excess metal ions for non-active binding sites^46^. Consistent with this, Cai et al. reported that EDTA increased keratinase activity from *Bacillus subtilis*^47^. In contrast, El-Gendy found that keratinases from *Penicillium* sp. Morsy1 were strongly inhibited by the chelating agents EDTA and EGTA^48^. These findings suggested that serine and cysteine residues are not involved in catalysis, supporting the classification of the enzyme as a metalloprotease. Similar to this study, Ramalingum et al. demonstrated that EDTA markedly reduced the keratinase activity of *Pseudomonas aeruginosa* S-04^49^.

Our results showed that β-mercaptoethanol markedly enhanced keratinase activity, indicating that the reducing agent disrupts disulfide bonds within the enzyme structure, thereby increasing catalytic efficiency. This stimulatory effect is consistent with reports demonstrating that reducing agents such as β-mercaptoethanol facilitate the cleavage of disulfide linkages and commonly enhance keratinase activity^50^. Similar observations were made for the keratinase produced by *Doratomyces microsporus*, where β-mercaptoethanol significantly increased enzyme activity^51^. In contrast, the keratinase from *Scopulariopsis brevicaulis* exhibited no change in activity in the presence of β-mercaptoethanol, highlighting species-specific variability in the response of keratinases to reducing agents^29^. Because keratin degradation requires both proteolysis and reduction of disulfide bonds, adding reducing agents effectively complements keratinase activity—explaining the dramatic increase observed in our data (e.g., > 1600% activity in the presence of 2-mercaptoethanol).

SDS markedly inhibited keratinase activity in this study, likely due to its strong protein-denaturing properties, which disrupt enzyme structure and catalytic function. Reports on keratinases that remain stable in the presence of SDS are limited. Gradišar et al.^52^ observed similar SDS-mediated inhibition in keratinases from *Paecilomyces marquandii* and *Doratomyces microsporus*. In contrast, the keratinase from *P. citrinum* AUMC 14742 showed enhanced activity when exposed to Tween-80, indicating that certain surfactants may promote substrate accessibility or stabilize the enzyme. Additionally, the keratinolytic serine protease from *Paecilomyces lilacinum* LPS#876 demonstrated stability in the presence of DMSO, methanol, Triton X-100, SDS, and Tween-80, further highlighting species-specific variability in surfactant tolerance^53^.

The optimization results also help explain the enzyme’s robust compatibility with a wide range of chemical additives. Enhancers such as Mn²⁺, Zn²⁺, Co²⁺, Tween-80, DMSO, and especially 2-mercaptoethanol—which increased activity by more than 1600%—reflect biochemical attributes consistent with an enzyme evolved to act on structurally rigid, disulfide-rich substrates like keratin. The strong activation by reducing agents was consistent with the mechanistic requirement of disulfide bond cleavage during keratin hydrolysis, while tolerance to detergents and organic solvents aligned with the surface-active, hydrophobic, and proteinaceous nature of the keratin substrate. These biochemical features amplified the relevance of the optimized production parameters: the statistical model favored conditions that stimulate secretion of a keratinase whose inherent properties make it exceptionally effective under alkaline, surfactant-rich, or redox-active industrial environments.

This study evaluated the dehairing efficiency of the crude keratinase produced by *P. citrinum* AUMC 14742 using goat skin, resulting in complete hair removal after 15 h at 30 °C. The leather industry commonly relies on toxic chemicals such as chromate and sulfide, which pose significant environmental hazards; therefore, microbial keratinase-based dehairing represents a safer and more sustainable alternative^54^. Keratinases play a critical role in improving leather quality by removing hair without damaging the underlying skin. Previous studies have shown that enzymatic treatment achieves superior results compared with chemical depilation, where sodium sulfite produces only partial hair removal, whereas keratinase enables complete dehairing within 12 h^55^. The keratinase from *P. citrinum* AUMC 14742 demonstrated competitive performance, achieving full dehairing in a time comparable to keratinases from *B. aerius* NSMk2^56^ and *B. cereus*, which required 15 and 12 h, respectively^4^. Sensory evaluation further revealed that enzymatically treated pelts exhibited improved organoleptic qualities, as the enzyme selectively degrades the non-structural proteinaceous material surrounding the hair root, facilitating scud removal and promoting fiber opening to yield a clean, wrinkle-free grain^57–59^.

The functional validation through goat-skin dehairing demonstrated the practical synergy between optimization and biochemical performance. Under the optimized production conditions, the crude keratinase preparation achieved complete dehairing within 15 h at 30 °C, confirming that the enzyme is not only efficiently produced but also highly effective in real-world applications under mild processing conditions. The rapid and non-destructive dehairing was directly attributable to the enzyme’s alkaline preference, thermo-stability, and compatibility with reducing conditions—properties that were elucidated through biochemical characterization but made operationally feasible through the optimization strategy.

## Materials and methods

### Fungal strain

During routine screening of *Penicillium* isolates from the Assiut University Mycological Centre collection, a highly efficient strain capable of rapidly hydrolyzing native chicken feathers was identified. This isolate was subsequently selected for detailed optimization of keratinase production in the present study.

### Morphological and molecular identification of the *Penicillium* isolate

Petri dishes containing Czapek’s agar (Cz), malt extract agar (MEA), and Czapek’s Yeast Autolysate agar (CYA) were used to cultivate the isolate AUMC 14742 of *Penicillium* used in this study^60^. The plates were then incubated at 25 °C for seven days. The *Penicillium* isolate AUMC 14742 was studied for its macroscopic and microscopic features. To molecularly confirm the strain identification, the PCR reaction was carried out at SolGent Co. (South Korea) using SolGent EF-Taq^61,62^. ITS region was amplified using the universal primers ITS1 and ITS4^63^.

### Extraction of keratin

The 500 g of native chicken feathers acquired from chicken farm (FM89 + Q78 2140001) in the Assiut Governorate of Egypt. The samples were taken from waste material collected from the ground. After being subjected to continuous agitation with a chloroform-methanol (1:1) solution for 24 h, the feather sample was defatted. Following this, it was washed three times with distilled water and finally oven-dried at 50 °C. Following the methods outlined in published works^6,22,23^, keratin was extracted from chicken feathers. The dried feathers were cut into small pieces (1–2 cm). 100 g of feather material were transferred into a 5000 mL Erlenmeyer flask. 1000 mL of 0.5 M NaOH was added, and the mixture was heated at 70 °C for 60 min with constant stirring to partially hydrolyze and soften the keratin. The slurry was allowed to cool to room temperature. The material was filtered through muslin cloth to remove undissolved debris. The filtrate was adjusted to pH 7.0 using 6 M HCl. Keratin in the neutralized solution was precipitated by adding four volumes of cold acetone with gentle agitation. The mixture was kept at 4 °C for overnight precipitation. The precipitated keratin was collected by centrifugation at 5000 rpm for 10 min. The pellet was washed twice with cold acetone to remove remaining salt, pigments, and lipids. After that, the extracted keratin was employed in the keratinase assay experiments.

### Fermentation medium and fermentation conditions

Sucrose-free Czapek’s broth supplemented with 1% native chicken feathers as the sole carbon source was used as the fermentation medium^6^. The medium (50 mL per 250 mL Erlenmeyer flask) was inoculated with 5 mL of a *Penicillium* cell suspension containing 1.5 × 10⁸ spores/mL obtained from a 7-day-old culture. The flasks were incubated at 25, 30, and 35 °C under shaking conditions (150 rpm) for seven days. After incubation, cultures were centrifuged at 10,000 rpm for 10 min at 4 °C, and the resulting cell-free supernatants were collected for use as the keratinase source in subsequent assays.

### Optimization of keratinase activity using Box-Behnken design (BBD)

In this experiment, three independent variables (temperature, pH, and peptone) were optimized using the Box-Behnken design (BBD) of response surface methodology (RSM). Each factor was evaluated at three coded levels (− 1, 0, + 1), and a total of fifteen experimental runs, including one center point, were generated using Design-Expert software. Point-prediction analysis was then applied to determine the optimal conditions for maximizing keratinase production (Table 7).

**Table 7:** Factors affecting keratinase production and their levels in Box-Behnken design BBD.

Independent variables	Symbol	Actual value of code		
		– 1	0	+ 1
Temperature	X1 = A	24	25	26
pH	X2 = B	7	8	9
Peptone (g/L)	X4 = D	1.5	2	2.5

The model underwent iterative methods, system identification, and parameter estimation to attain improved accuracy and dependable simulation results. The second-order polynomial equation produced is presented in Eq. (2):

2 $$Y=\beta_o+\Sigma\beta_iX_i+\Sigma \beta_{ij}X_iX_j+\Sigma\beta_{ii}X_i^2$$

Where Y is the predicted response, β_0_ is the model constant; X_i_ are independent factors (temperature, pH, and peptone); β_i_ and β_ii_ are coefficients. To determine the significance of the model terms, the data obtained from the (BBD) were subjected to ANOVA analysis. Following the optimization of keratinase production using the Box–Behnken design, it was essential to evaluate the reliability of the predicted model under experimental conditions. Therefore, a validation step was conducted to confirm that the optimized parameters not only improved production statistically but also translated into reproducible enzymatic yields.

### Keratinase assay

A 1.0 mL of the fungal supernatant was mixed with 0.01 g of keratin (prepared in 1.0 mL of phosphate buffer, pH 8.0) and incubated for 60 min at 50 °C. The reaction was then terminated by adding 2.0 mL of 10% trichloroacetic acid (TCA). After centrifugation at 10,000 rpm for 10 min at 4 °C, the precipitate was removed. A 0.2 mL aliquot of the resulting supernatant was diluted to 1.0 mL, followed by the addition of 5 mL of alkaline copper reagent. The mixture was kept in the dark for 30 min and subsequently treated with 0.5 mL of Folin–Ciocalteu reagent to develop the blue color, which was measured at 660 nm^6^. Keratinase activity was calculated using a tyrosine calibration curve, where one unit of activity corresponds to the amount of enzyme that releases 1 µmol of tyrosine per minute.

Having confirmed the accuracy and robustness of the optimized conditions through validation experiments, the study then progressed to purifying and characterizing the keratinase to better understand its biochemical properties and industrial potential. This transition from optimization to molecular characterization allowed for linking production efficiency with functional enzyme performance.

### Purification of keratinase

#### Precipitation by absolute ethyl alcohol

Upon reaching peak activity under the optimized growth conditions, the culture supernatant was collected by centrifugation at 10,000 rpm for 10 min at 4 °C. The clarified supernatant was then slowly mixed with cold absolute ethyl alcohol (− 25 °C) under gentle agitation at 4 °C to facilitate protein precipitation. The resulting precipitate was subsequently isolated, lyophilized, and subjected to further purification.

#### Lewatit MonoPlus (MP 800) anion exchange column

With a bed capacity of 200 cm³, pre-activated MP 800 gel^6^ was loaded into a glass column (60 cm × 2.4 cm). The ion-exchange column was loaded with 10 mL of the crude keratinase preparation. Elution was performed using a 100 mM phosphate buffer (pH 7.0) combined with a linear NaCl gradient ranging from 0 to 1.5 M at a flow rate of 0.5 mL/min. Fractions of 6.0 mL showing the highest enzyme activity and protein content were pooled, concentrated, and used for subsequent purification steps.

#### Sephadex G50 gel size exclusion column

A 10 mL aliquot of the enzyme preparation was applied to a Sephadex G-50 gel filtration column (60 cm × 2.4 cm) for further purification. Proteins were eluted using 100 mM phosphate buffer (pH 7.0) at a flow rate of 0.5 mL/min. The fractions exhibiting the highest enzymatic activity were pooled, concentrated, and used for subsequent characterization analyses.

#### SDS-PAGE

SDS–PAGE was performed using a discontinuous buffer system composed of a 12% (w/v) polyacrylamide resolving gel and a 4% stacking gel. Protein samples (crude or purified) were prepared in 300 µL of RIPA lysis buffer containing 1 µg/mL leupeptin and aprotinin, 0.5 mM PMSF, 1 mM NaOH, and 5 mM sodium fluoride. After protein concentrations were determined using the Bradford assay^64^, sample buffer was added and the samples were boiled for 5 min. Each well was loaded with 30 µL of sample (approximately 30 µg/mL protein) along with 5 µL of a pre-stained protein marker. Electrophoresis was carried out at 60 V for the stacking gel and 120 V for the resolving gel under cooling. Following separation, the gel was stained with Coomassie Brilliant Blue R-250 for 30 min and left to destain overnight. The gel was scanned, and the Rf values of the marker proteins and sample bands were calculated using ImageJ software. A standard curve of Rf versus the logarithm of molecular weight was constructed, and the apparent molecular weight of the target protein was determined by interpolating its corresponding Rf value.

With a purified enzyme preparation in hand, subsequent assays were designed to characterize its catalytic behavior under different physicochemical conditions. These biochemical analyses provided critical insights into the enzyme’s stability, activity profile, and compatibility with industrial environments.

#### Impact of pH and temperature on the activity of pure keratinase

The ideal pH for keratinase was determined by first incubating 0.01 g of purified enzyme and 0.01 g of pure keratin (dissolved in 1.0 mL of buffer solution; pH 5–11) at 45 °C. When tested at the enzyme’s ideal pH, pure keratinase displayed activity over a temperature range of 30–65 °C.

#### Ions and inhibitors’ effects on the activity of pure keratinase

The effects of various organic solvents, including DMSO, hexane, chloroform, benzene, acetone, methanol, and ethanol, were studied under optimal pH and temperature settings. The solvent concentrations tested were 5% and 10%. The following metal ions were tested: Na^+^, K^+^, Ca^2+^, Co^2+^, Ni^2+^, Cu^2+^, Fe^2+^, Mn^2+^, Mg^2+^, and Zn^2+^; these ions were administered as NaCl, KCl, CaCl_2_, CoCl_2_, NiCl_2_, CuSO_4_, FeSO_4_, MnSO_4_, MgSO_4_, and ZnSO_4_ at concentrations of 5 mM ^6,23^. The effect of sodium dodecyl sulphate (SDS), EDTA, and phenylmethylsulphonyl fluoride (PMSF) on keratinase activity was determined at 5 mM concentration of each. 2-Mercaptoethanol and 2-Dimethylsulfoxide (DMSO) were also tested at concentrations of 5 and 10% each. For comparison, the enzyme activity in the absence of any additive was considered as 100% (Control activity). The activity measured in the presence of each metal ion, inhibitor, solvent, or surfactant was expressed as residual activity (%), calculated as: (activity with additive/control activity) × 100. This format allows clearer visualization of the relative impact of each compound on keratinase stability.

#### Determination of K_m_ and V_max_

The kinetic constants Km and Vmax for the keratinase activity were calculated by conducting a pure keratin experiment with substrate concentrations ranging from 10 to 100 mg/mL^65^.

The detailed biochemical characterization established the enzyme’s robustness and suitability for practical applications. To translate these findings into a real-world context, keratinase was further evaluated for its functional efficiency in goat-skin dehairing, serving as a direct demonstration of its applied potential.

#### Dehairing activity

The effectiveness of *P. citrinum* AUMC 14742 crude keratinase in removing epidermal hairs was evaluated using goat skin obtained from a local slaughterhouse in Assiut Governorate, Egypt. The goat skin was thoroughly cleansed using both tap and distilled water to remove dirt and blood. Employing the approach described by^6,66^, samples of goat skin (5 cm × 10 cm) were individually submerged in 100 mL of the crude keratinase preparation of *P. citrinum* AUMC 14742 at a concentration of 312 U/mL, and thereafter maintained at 30 °C. After incubation, the goat skin was taken out from the supernatant at five-hours intervals and thoroughly cleaned with distilled water. The dehairing experiment lasted for 48 h.

### Statistical analysis

All experiments were conducted in triplicate, and the mean and standard deviation was used to express all data. The statistical significance analysis approach of Stahle and Wold^67^ was applied. It was deemed significant at *p* ≤ 0.05.

## Conclusion

This work provides the first comprehensive demonstration that *Penicillium citrinum* AUMC 14742 is an exceptionally potent source of alkaline keratinase with characteristics distinctly superior to previously reported fungal and bacterial keratinases. Unlike earlier studies that only documented crude feather degradation by *P. citrinum*, this investigation delivers a complete and rigorously optimized production–purification–characterization pipeline. Through Box–Behnken design, keratinase synthesis was significantly improved, yielding one of the highest keratinase activities reported for any *Penicillium* species. The multi-step purification strategy produced a homogeneous enzyme with a remarkable 143-fold increase in purity and a specific activity of 35,423 U/mg—values not previously documented for *P. citrinum*. Critically, this is the first study to reveal that the enzyme possesses a unique combination of industrially desirable traits: strong alkalophilicity (pH 10), thermostability (55 °C), exceptional tolerance to detergents and organic solvents, and unprecedented activation by Mn²⁺ and reducing agents. The keratinase also displayed high catalytic efficiency, with kinetic parameters confirming its strong substrate affinity. Most importantly, the enzyme’s real-world relevance was validated for the first time by achieving complete enzymatic dehairing of goat skin within 15 h at 30 °C, firmly establishing its superiority as a green alternative to hazardous chemical unhairing agents. Together, these findings introduce *P. citrinum* AUMC 14742 as a novel, high-performance producer of an alkaline, thermo-stable, and chemically resilient keratinase with direct applicability in leather processing, detergents, and feather waste valorization. The study fills a major knowledge gap by providing the first detailed biochemical framework for *P. citrinum* keratinase and reveals its significant promise as a next-generation biocatalyst for sustainable industrial biotechnology.

## Data Availability

The dataset generated and/or analyzed during the current study is available in the GenBank: [*Penicillium citrinum strain AUMC 14742 internal transcribed spacer 1, - Nucleotide - NCBI*](https:/www.ncbi.nlm.nih.gov/nucleotide/PV069363.1?report=genbank&log$=nucltop&blast_rank=8&RID=KSU81DYU016) ; accession number PV069363.
